# A novel system for tracking social preference dynamics in mice reveals sex- and strain-specific characteristics

**DOI:** 10.1186/s13229-017-0169-1

**Published:** 2017-10-03

**Authors:** Shai Netser, Shani Haskal, Hen Magalnik, Shlomo Wagner

**Affiliations:** Sagol Department of Neurobiology, Faculty of Natural Sciences, University of Haifa, 199 Aba Khushi Ave. Mt. Carmel, 3498838 Haifa, Israel

**Keywords:** Social preference, Social investigation, Three-chamber test

## Abstract

**Background:**

Deciphering the biological mechanisms underlying social behavior in animal models requires standard behavioral paradigms that can be unbiasedly employed in an observer- and laboratory-independent manner. During the past decade, the three-chamber test has become such a standard paradigm used to evaluate social preference (sociability) and social novelty preference in mice. This test suffers from several caveats, including its reliance on spatial navigation skills and negligence of behavioral dynamics.

**Methods:**

Here, we present a novel experimental apparatus and an automated analysis system which offer an alternative to the three-chamber test while solving the aforementioned caveats. The custom-made apparatus is simple for production, and the analysis system is publically available as an open-source software, enabling its free use. We used this system to compare the dynamics of social behavior during the social preference and social novelty preference tests between male and female C57BL/6J mice.

**Results:**

We found that in both tests, male mice keep their preference towards one of the stimuli for longer periods than females. We then employed our system to define several new parameters of social behavioral dynamics in mice and revealed that social preference behavior is segregated in time into two distinct phases. An early exploration phase, characterized by high rate of transitions between stimuli and short bouts of stimulus investigation, is followed by an interaction phase with low transition rate and prolonged interactions, mainly with the preferred stimulus. Finally, we compared the dynamics of social behavior between C57BL/6J and BTBR male mice, the latter of which are considered as asocial strain serving as a model for autism spectrum disorder. We found that BTBR mice (*n* = 8) showed a specific deficit in transition from the exploration phase to the interaction phase in the social preference test, suggesting a reduced tendency towards social interaction.

**Conclusions:**

We successfully employed our new experimental system to unravel previously unidentified sex- and strain-specific differences in the dynamics of social behavior in mice. Thus, the system presented here facilitates a more thorough and detailed analysis of social behavior in small rodent models, enabling a better comparison between strains and treatments.

**Electronic supplementary material:**

The online version of this article (10.1186/s13229-017-0169-1) contains supplementary material, which is available to authorized users.

## Background

Unraveling the biological mechanisms underlying pathological conditions characterized by atypical social behavior, such as autism spectrum disorder (ASD), is currently one of the main challenges in the field of social neuroscience [1]. Addressing this challenge requires the use of standard behavioral paradigms that typify the behavior of animal models in an unbiased way. In a seminal work published more than a decade ago, Moy and colleagues [2] presented the three-chamber test, which has become a standard way to evaluate social behavior in animal models of ASD.

This paradigm is mostly used to assess how much a rodent subject prefers a social stimulus over an object one (social preference (SP), also termed sociability), as well as how much it prefers a novel social stimulus over a familiar one (social novelty preference (SNP)), which is the innate tendency of mice and rats [3, 4]. The test, based on measuring the time spent by the subject in either a central chamber or each of two lateral chambers where distinct stimuli are located, suffers from several caveats. First, it largely depends on the preference of the subject to locate itself in one of the chambers, hence should be sensitive to parameters that influence spatial navigation, memory, and preference. Under certain conditions, such parameters may vary independently of the motivation of the subject for a direct social interaction and thereby interfere with the efficiency of the test to directly measure social motivation and preference. Second, this test is mostly used to measure the total time spent by the subject in each of the chambers, while neglecting the behavioral dynamics.

Here, we present a novel experimental apparatus and automated analysis system that offer an alternative to the three-chamber test and enable performing the same behavioral examinations while solving the aforementioned caveats. The custom-made apparatus is simple for production, and the analysis system is publically available as an open-source program, thereby allowing any lab to easily employ it. We demonstrate the ability of this system to measure novel parameters of murine social behavior, thus to detect previously unidentified sex- and strain-specific differences in the dynamics of social preference and social novelty preference.

## Results

### The experimental system

**Fig. 1:**
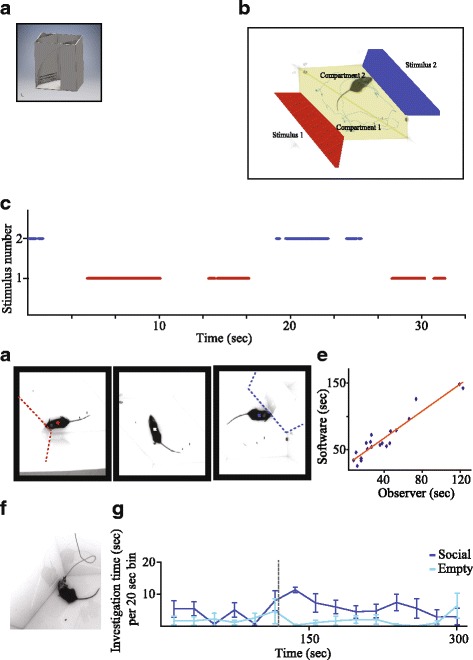
The experimental setup and analysis system. **a** The experimental arena, pictured from above with a mouse exploring two social stimuli located in grooved chambers. Inset—schematic representation of the experimental setup with grooved chambers. **b** Computer screenshot during analysis, showing the arena viewed from above. The front walls of the triangular chambers are marked as “stimulus 1” and “stimulus 2,” and the arena floor is divided into “compartment 1” and “compartment 2.” Also, shown are the tracked mouse and the tracked path. **c** Plot of investigation bouts during 35 s of the experiment shown in **b**, labeled according to the number of investigated stimulus (red for stimulus 1, blue for stimulus 2). **d** Four frames from the video file of the experiments shown in **b**, demonstrating distinct behavioral events and the location of the center of the animal body (cross) identified by the analysis system for each one. The cross is white during non-investigative behavior, red during investigation of stimulus 1, and blue during investigation of stimulus 2. Dashed lines mark the borders of the chambers. **e** Analysis of the correlation between investigation times of both object and social stimuli measured by a trained observer during SP experiments (*n* = 20 male mice) and those measured for the same experiments by the computerized analysis system. **f** Representative image of a mouse with an electrical wire connecting head-implanted probe to electrophysiological recording system in the experimental arena. **g** Analysis of mean (±SEM) investigation time of wired animals (*n* = 4) towards two chambers located in opposite corners of the arena while both were empty (before the time marked by dashed line) and after insertion of a social stimulus to one of them (after the time marked by dashed line)

The experimental setup (described in detail in the “Methods” section and depicted in Fig. 1a) consists of a white Plexiglass arena with two Plexiglass triangular chambers randomly located at its opposite corners, placed at the middle of an acoustic cabinet. Two versions of triangular chambers were used, one (grooved chambers) with horizontal slots allowing restricted access to the stimulus and the other (meshed chambers) with a metal mesh, allowing direct interaction with the stimulus.

Video files of the experiments were later analyzed using custom-made software, written in MATLAB (body-based algorithm, see graphical user interface in Additional file 1) that automatically and continuously tracks subject location, based on its body center, as well as its contact with the stimuli-containing chambers. The analysis procedure is as follows: after uploading a movie file, the experimenter graphically defines two areas as “compartments” and two additional areas as “stimuli” (Fig. 1b). The software detects the video frames in which the subject is located in areas defined as “compartments” and calculates the time spent in each one. To determine investigation of stimuli, the software tracks contact between areas defined as “stimuli” with the body borders of the subject (Fig. 1c). Such contact events (Fig. 1d, see video movie in Additional file 2) are defined as investigatory and serve for calculating the investigation time. Although the algorithm is simple, we found an excellent correlation (*r* ^2^ = 0.91, *p* < 10^−6^, Pearson’s correlation) between the investigation time measured manually by an observer and by the software (Fig. 1e). In the software interface, the parameters for analysis can be defined and uploaded for a batch of movie files and the results from several animals can be pooled and averaged for further analysis.

To examine the possibility that a significant error is introduced due to non-investigatory contact events between the stimuli and the body borders of the subjects, we used another algorithm (head directionality-based algorithm) which adds head directionality to the subject and measured contacts with the head only (see video movie in Additional file 3). We compared the investigation time data obtained with the head directionality-based algorithm, which is much slower, hence not practical for analysis of large datasets, with those obtained with the body-based algorithm, and found very similar results (1.1% difference, *n* = 11 experiments of 5 min each, not shown). Thus, we used the basic body-based algorithm for analysis of the results throughout the current study.

Finally, we evaluated the ability of our system to analyze the investigation behavior of mice connected with cables containing electrical wires or optical fibers, as may be needed for recording of brain activity or optogenetic stimulation. To that end, we developed a modified algorithm (wired body-based algorithm, see the “Methods” section) that tolerates the cable connected to the mouse head. Using this algorithm, the system could track the instigation behavior of a male mouse performing the SP test while the mouse was connected to a cable (Fig. 1f). The mean results of four such experiments with distinct animals are displayed in Fig. 1g. As apparent, the animals showed no preference towards any of the empty chambers but did show social preference after the introduction of a social stimulus to one of them (introduction time marked by a dashed line).

### Comparison between male and female mice

**Fig. 2:**
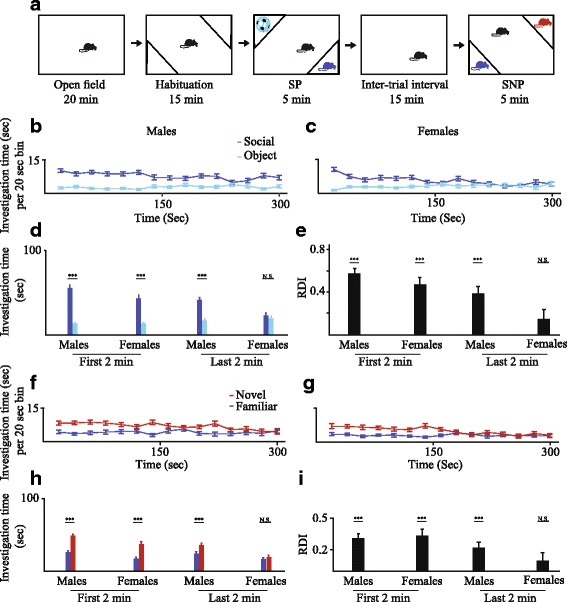
Different behavioral dynamics between male and female mice. **a** Schematic representation of the experimental paradigm, combining sequential open field, social preference (SP), and social novelty preference (SNP) tests. **b** Plot of mean (±SEM) investigation time (measured within 20-s bins) across the SP session for male mice (*n* = 33). **c** As in **b**, for female mice (*n* = 29). Note the loss of preference after 150 s. **d** Mean investigation time for both stimuli during the first (left) and last (right) 2 min of the SP experiments. All categories of analysis yielded significant preference of the social stimulus (one-tailed paired *t* test) except for the last 2 min in females. **e** Mean ratio of duration of investigation (RDI) of object stimuli to social ones for all categories shown in **f**. All categories yielded a significant difference from zero (post hoc *t* test following main effects of both time and sex, MM-ANOVA), except for the last 2 min in females. **f** Plot of mean (±SEM) investigation time (measured within 20-s bins) across the SNP session for male mice (*n* = 33). **g** As in **b**, for female mice (*n* = 29). Note the loss of preference after 150 s. **h** Mean investigation time for both stimuli during the first (left) and last (right) 2 min of the SNP experiments. All categories of analysis yielded significant preference of the novel social stimulus (one-tailed paired *t* test) except for the last 2 min in females. **i** Mean ratio of duration of investigation (RDI) of familiar stimuli to novel ones for all categories shown in **e**. All categories yielded a significant difference from zero (post hoc *t* test following main effect of time, MM-ANOVA), except for the last 2 min in females. ***p < 0.001

We first examined whether we can perform a paradigm similar to the three-chamber test with our system. Thus, we quantified, using grooved chambers, the behavior of subjects in sequential open field, SP, and SNP tasks (see Fig. 2a for a scheme of the paradigm) of adult male and female C57BL/6J mice (33 males and 29 females, 3 groups of 8–15 animals per sex). In the open-field test, we found a significant difference between males and females in the total distance traveled (males 25.8 ± 0.25 m, females 20.58 ± 1.68 m, mean ± SEM, *p* < 0.01, two-tailed *t* test).

We then compared the male and female mice in the SP test (Fig. 2a). As displayed in Fig. 2b, c, the preference to investigate the social stimulus over the object decreased with time for both sexes. However, this decrease was more profound in the case of female mice, which did not seem to show any preference during the last 2 min of the test, while males kept a clear preference towards the social stimulus throughout the 5-min test. A similar conclusion could be drawn from the analysis of time spent in the different compartments (Additional file 4A, B). It should be noted that these results were highly repeatable between experimental groups, as exemplified in Additional file 5 which depicts the results of two experimental groups for each sex.

Accordingly, when the mean investigation time was analyzed separately for the first and last 2 min of the test, statistically significant preference for the social stimulus over the object was obtained for males in both periods, while females showed such preference only in the early period (Fig. 2d; *p* < 0.001, one-tailed paired *t* test). To directly compare between males and females, we calculated the relative duration of investigation (RDI, see the “Methods” section) of object stimuli to social ones for each animal (Fig. 2e). Statistical comparison between sexes and periods performed on the RDI values revealed a significant main effect of both sex and period with no interaction between the two (sex: *F*_1,60_ = 6.21, *p* = 0.015; period: *F*_1,60_ = 11.49, *p* = 0.001; interaction: *F*_1,60_ = 0.31, *p* = 0.57; MM-ANOVA). In agreement with the *t* test performed on investigation time, only females during the last 2 min did not show a significant difference from zero (post hoc *t* test, *p* < 0.001 for all comparisons except females during the last 2 min, where *p* = 0.062). Thus, while social preference declines with time in both sexes, it is more persistent in male mice.

Qualitatively, similar results were found in the SNP test. As demonstrated in Fig. 2f, g for investigation behavior (Additional file 4C, D for time spent in the different compartments), both male and female mice showed clear preference towards the novel social stimulus during the early stage of the test. Yet, during the later phase, female mice did not show such preference while male mice did (Fig. 2h; *p* < 0.001, one-tailed paired *t* test).

Statistical comparison between sexes and periods performed on the RDI of familiar to novel stimuli (Fig. 2i) revealed a significant main effect of period but not sex with no interaction between the two (sex: *F*_1,60_ = 0.51, *p* = 0.47; period: *F*_1,60_ = 7.67, *p* = 0.007; interaction: *F*_1,60_ = 1.58, *p* = 0.21; MM-ANOVA). In agreement with the *t* test performed on investigation time, only females during the last 2 min did not show a significant difference from zero (post hoc *t* test, *p* < 0.001 for all comparisons except females during the last 2 min, where *p* = 0.204). We conclude that in both sexes, there is a significant decline of SNP following the first 2 min of test. Yet, this effect is more pronounced in females, which exhibit a complete loss of SNP during the last 2 min. Besides this difference in behavioral dynamics, male mice exhibited significantly higher total social investigation time (of both stimuli) than females (males 170.9 ± 6.13 s; females 117.2 ± 7.88 s, *p* < 0.001, two-tailed *t* test). Thus, using our system, we revealed novel sex-dependent differences in the dynamics of murine social behavior in the SP and SNP tests.

### Comparison between meshed and grooved chambers

**Fig. 3:**
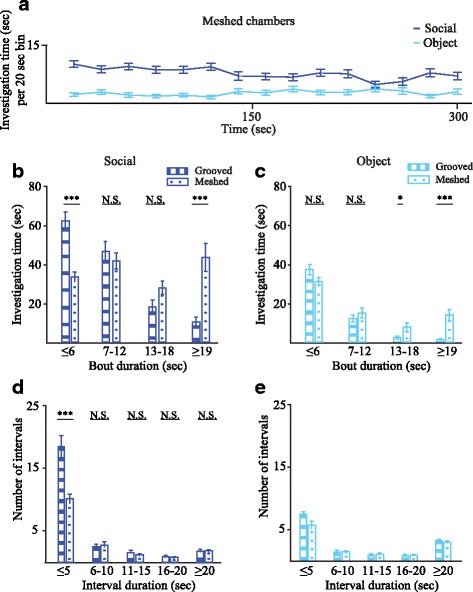
Meshed chambers enhance social interactions as compared to grooved chambers. **a** Plot of mean investigation time (measured within 20-s bins) across the SP session for male mice (*n* = 45) using meshed chambers. **b**, **c** Distribution of time dedicated for investigating social (**b**) and object (**c**) stimuli during SP experiments with grooved (*n* = 33) and meshed (*n* = 45) chambers, according to bout duration (post hoc *t* test following interaction, MM-ANOVA). **d**, **e** Distribution of the number of intervals between repeated investigations of social (**d**) and object (**e**) stimuli during SP experiments with grooved and meshed chambers, according to interval duration (post hoc *t* test following interaction, MM-ANOVA). ***p < 0.001, *p < 0.05

We next examined whether the use of meshed chambers, which enable more direct interactions between the subject and stimuli, yields similar results to the grooved chambers used so far. We therefore performed the same behavioral paradigm described in Fig. 2a with a new cohort of male mice (*n* = 45, three groups of 15 animals) using the meshed chambers described in the “Methods” section. It should be noted that a much smaller group size is needed for observing a preference between the two stimuli in our system, as exemplified in Additional file 5. Power calculations reveal that sample sizes of five and eight animals are required for the SP and SNP tests, respectively (*α* = 0.05, power = 0.8). Yet, we used a significantly bigger group size in order to make sure we do not miss subtle differences in the dynamics of social behavior. As shown in Fig. 3a, the investigation times measured with the meshed chambers during the SP test seem very similar to those obtained with grooved chambers (Fig. 2b). We therefore examined two parameters that characterize the type of interactions between the subject and the stimuli. First, we measured the duration of each investigation bout and compared the distribution of investigation time according to this parameter between the two chamber types, separately for each stimulus (Fig. 3b). We found a statistically significant interaction between the type of chambers and the duration of social investigation bouts (F_2.436,180.242_ = 16.366, *p* < 0.001; MM-ANOVA). Specifically, while with the grooved chambers, we observed more time used for short (≤ 6 s) social investigation bouts; the meshed chambers were characterized by more time dedicated for long (≥ 19 s) bouts (post hoc *t* test, *p* < 0.001). Similar but much smaller difference was observed for the object stimulus (Fig. 3c; F_2.382,176.254_ = 7.744, *p* < 0.001, MM-ANOVA). Second, we measured the intervals between consecutive investigations of the same stimulus and categorized them according to their length. This analysis demonstrated a statistically significant interaction between chamber type and interval duration (*F*_1.306,96.626_ = 17.613, *p* < 0.001, MM-ANOVA), with more short intervals observed with the grooved as compared to the meshed chambers for social stimuli (Fig. 3d, post hoc *t* test, *p* < 0.001), while no such difference was found for the objects (Fig. 3e, type: *F*_1,74_ = 0.972, *p* = 0.327; duration: *F*_1.909,141.266_ = 95.061, *p* < 0.001; interaction: *F*_1.909,141.266_ = 1.518, *p* = 0.223). Altogether, these analyses suggest that meshed chambers, which allow better interaction between the animals, promote extended social investigation bouts, as compared to the grooved chambers which enhance fragmented interactions with the social stimulus.

### The dynamics of social preference and social novelty preference

**Fig. 4:**
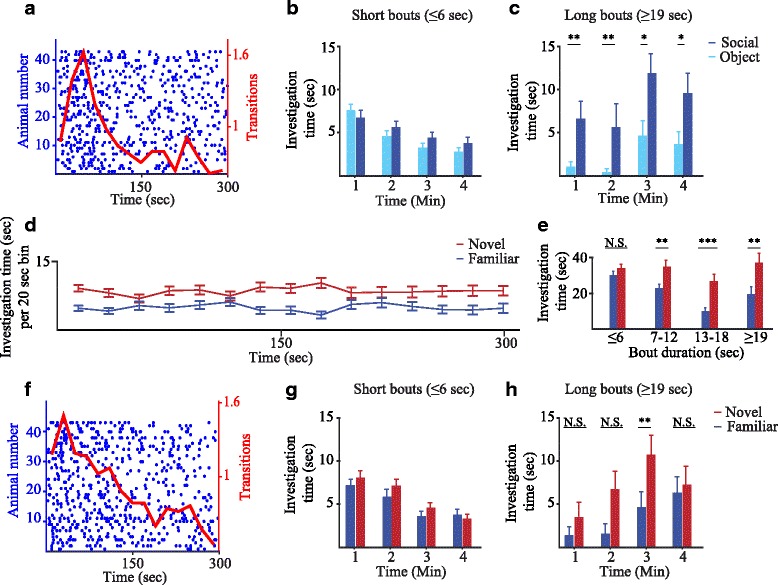
Distinct phases of social behavior in the SP and SNP tests. **a** Transitions, represented by blue dots, between stimuli during the SP test. Each line represents one mouse (45 males), and the red line represents the mean of all mice averaged within 20-s bins. **b** Investigation time (mean + SEM within 1-min bins) across the SP test when short bouts (< 6 s) are separately analyzed. **c** As in **b**, for long investigation bouts (> 19 s). A significant difference was found between social and object stimuli (post hoc *t* test following main effect). **d** Plot of mean investigation time (measured within 20-s bins) across the SNP session for male mice (*n* = 45) using meshed chambers. **e** Distribution of mean total investigation time (+SEM) according to bout duration, for both social stimuli. A significant difference between the familiar and novel stimuli was observed only for bouts which are longer than 6 s (post hoc *t* test following main effect). **f** As in **a**, for the SNP test. **g** As in **b**, for the SNP test. **h** As in **c**, for the SNP test. A significant difference was found between the stimuli (post hoc *t* test following main effect)

We then used the meshed chambers to explore the dynamics of stimuli investigation in the SP test. We started with plotting the time points when the subject started a fresh investigation of a stimulus (after returning from investigating the other stimulus). This parameter thus represents transitions between stimuli. As apparent in Fig. 4a, the mean number of transitions rapidly rises to a clear peak at 50 s after the beginning of the test and then decays to about 25% of the peak value at 150 s. We then examined the change in length of investigation bouts during the test. We found that while the time dedicated for short bouts (< 6 s) gradually decreased by > 50% during the test for both stimuli (Fig. 4b) with no difference between them (stimulus: *F*_1,42_ = 1.099, *p* = 0.3; period: *F*_2.695,113.174_ = 16.189, *p* < 0.001; interaction: *F*_2.766,116.181_ = 1.133, *p* = 0.337, TWR-ANOVA), the time spent on long bouts (> 19 s) was doubled between the first and third minutes of the test (Fig. 4c), and most of this time was dedicated for social investigation (stimulus: *F*_1,42_ = 14.385, *p* < 0.001; period: *F*_3,126_ = 3.785, *p* = 0.012; interaction: *F*_3,126_ = 0.141, *p* = 0.935, TWR-ANOVA). These results suggest an interesting and novel dynamics of the social preference test; the first 2 min, termed by us the exploration phase, are characterized by a large number of transitions between stimuli and short investigation bouts, while the rest of test, termed by us the interaction phase, is characterized by a lower number of transitions and longer bouts of interaction with stimuli, mainly with the social stimulus.

Very similar results were obtained when the SNP test was performed with male mice using meshed chambers. As with grooved chambers (Fig. 2f), clear preference towards the novel stimulus was observed throughout the test (Fig. 4d), with most of the difference attributed to longer bouts (Fig. 4e, stimulus: *F*_1,42_ = 17.692, *p* < 0.001; duration: *F*_2.057,86.385_ = 4.9, *p* = 0.009, interaction: *F*_2.373,99.674_ = 2.222, *p* = 0.104, TWR-ANOVA). The dynamics of the bouts was also very similar to that described above for the SP test, with early exploration phase characterized with high level of transitions between stimuli (Fig. 4f) as well as short bouts that did not differ between stimuli (Fig. 4g, stimulus: *F*_1,42_ = 1.59, *p* = 0.214; duration: *F*_2.590,108.771_ = 22.271, *p* < 0.001; interaction: *F*_2.739,115.043_ = 0.695, *p* = 0.544, TWR-ANOVA). In contrast, the late interaction phase (last 3 min of the test) was characterized with low level of transitions and more investigation time dedicated to long bouts, mostly with the novel stimulus (Fig. 4h, stimulus: *F*_1,42_ = 8.672, *p* = 0.005; duration: *F*_2.603,109.347_ = 6.899, *p* = 0.001; interaction: *F*_2.808,117.919_ = 1.861, *p* = 0.144, TWR-ANOVA), suggesting a similar division of the SNP test into exploration and interaction phases.

### Exploring the parameters of social behavioral dynamics

**Fig. 5:**
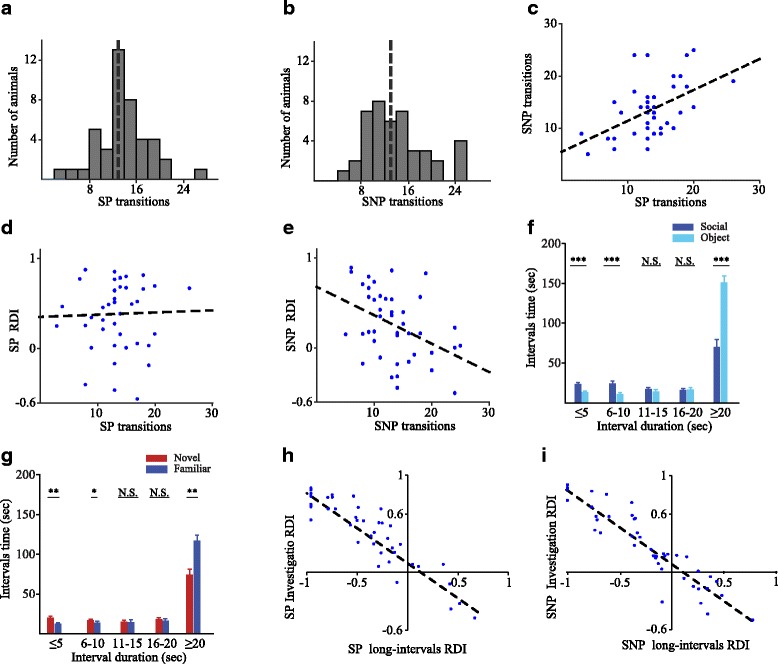
Parameters of behavioral dynamics in the SP and SNP tests. **a** Distribution of the tested mice according to the total number of transitions made by each subject in the SP test. Dashed line marks the mean value. **b** As in **a**, for the SNP test. **c** A significant positive correlation (*r* = 0.51, *p* < 0.001, Pearson’s correlation) was found between the number of transitions made by each subject during the SP and SNP tests. **d** No correlation was found between the RDI and total number of transitions during the SP test. **e** A significant negative correlation (*r* = − 0.42, *p* < 0.01) was found between the RDI and total number of transitions during the SNP test, suggesting a link between the number of transitions made by the subject and its recognition of the familiar social stimulus. **f** Distribution of the total time spent by the subject during the SP test on intervals between consecutive investigation bouts directed towards each stimulus, according to the length of interval. **g** As in **f**, for the SNP test. **h** A significant negative correlation (*r* = − 0.9, *p* < 0.05) was found between the RDI calculated according to the difference in investigation time (higher for the preferred stimulus) and the RDI calculated according to the difference in prolonged intervals (lower for the preferred stimulus, in the SP test). **i** As in **h**, for the SNP test (*r* = − 0.92, *p* < 0.001). ***p < 0.001, **p < 0.01, *p < 0.05

We then explored the new parameters that were found by us to describe the dynamics of social behavior. We started by comparing the distribution of the total number of transitions made by each subject between the SP and SNP tests. As apparent in Fig. 5a, b, the total number of transitions was normally distributed in both SP and SNP tests, with most (74 and 63%, respectively) cases located within the range of 10–20 transitions. Yet, the SP distribution was more narrow (13.46 ± 4.34) while the SNP distribution had the same mean but was wider (13.46 ± 5.07), with 60% of the cases evenly distributed between 8 and 16, suggesting a tighter tendency for certain exploration level during the SP test. Nevertheless, we found a statistically significant positive correlation (*r* = 0.51, *p* < 0.001, Pearson’s correlation) between the total number of transitions made by each individual mouse in each test (Fig. 5c). This correlation suggests that the number of transitions reflects a characteristic exploration tendency of the individual subject. We then examined if there is a correlation between the number of transitions made by each subject and its RDI value in the SP and SNP tests. Interestingly, while no such correlation was revealed for the SP test (Fig. 5d; *r* = 0.002, *p* = 0.84), we found a statistically significant negative correlation between these parameters in the SNP test (Fig. 5e; *r* = − 0.42, *p* < 0.01), suggesting that a large number of transitions reflects a difficulty in social recognition. The lack of correlation between transitions and RDI in the SP test, as compared to the significant negative correlation between the same parameters in the SNP test, suggests that the two tests detect distinct aspects of social behavior which are characterized by different relationships between the parameters describing the social behavioral dynamics.

We also examined the intervals between consecutive investigations of the same stimulus by plotting the mean total time of intervals as a function of the interval duration. We found a statistically significant interaction between the stimulus and interval duration in both the SP (Fig. 5f) and SNP (Fig. 5g) tests (SP: *F*_1.294,54.347_ = 28.352, *p* < 0.001; SNP: *F*_1.316,55.275_ = 6.723, *p* = 0.007, TWR-ANOVA). Specifically, the preferred stimulus was characterized by more time spent on short (< 10 s) intervals and less time spent on long (> 20 s) intervals (post hoc *t* test, *p* < 0.001). Moreover, there was a significant negative correlation between the RDI calculated according to the difference in investigation time (higher for the preferred stimulus) and the RDI calculated according to the difference in prolonged intervals (lower for the preferred stimulus), in both the SP (Fig. 5h; *r* = − 0.9, *p* < 0.05) and SNP (Fig. 5i; *r* = − 0.92, *p* < 0.001) tests. Thus, the difference in prolonged intervals may be used to assess preference between stimuli in the SP and SNP tests independently of the investigation time.

### The dynamics of social preference of BTBR mice

**Fig. 6:**
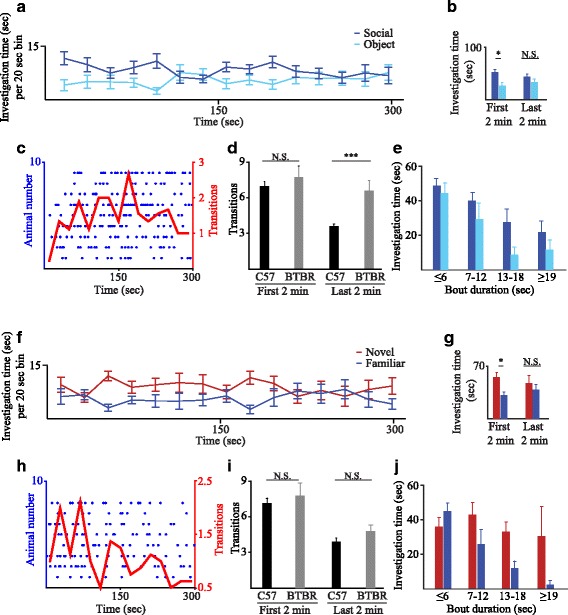
Modified dynamics of social preference in BTBR mice. **a** Plot of mean investigation time (measured within 20-s bins) across the SP test for male BTBR mice (*n* = 8) using meshed chambers. **b** Mean (+SEM) investigation time averaged across the first (left) and last (right) 2 min of the SP test, showing a significant preference only during the early 2 min (*p* < 0.05 paired-test after Bonferroni correction). **c** Transitions, represented by blue dots, between stimuli during the SP test. Each line represents one BTBR mouse, and the red line represents the mean of all mice averaged within 20-s bins. **d** A comparison of mean number of transitions for the first and last 2 min of the SP test, between BTBR and C57BL/6J male mice (*p* < 0.05, post hoc *t* test following a main effect). **e** Distributions of mean total investigation time (+SEM) according to bout duration, for social and object stimuli during the SP test performed by BTBR male mice. **f**–**j** As in **a**–**e**, respectively, for the SNP test. ***p < 0.001, *p < 0.05

We then examined if our system can be used in order to reveal and analyze strain-dependent differences in social behavior. To that end, we explored the dynamics of the SP and SNP tests in BTBR mice, which show atypical social behavior and are considered as a model for ASD [5]. As apparent in Fig. 6a, b, BTBR male mice showed social preference which was weaker and less persistent as compared to C57BL/6J male mice (Fig. 2b), with a significant social preference only during the first 2 min (Fig. 6b, *p* < 0.05 paired *t* test after Bonferroni correction), thus resembling the behavior of C57BL/6J female mice (Fig. 2c). Moreover, when the transitions dynamics was analyzed, we found that the mean number of transitions exhibited by the BTBR mice is kept much higher than that of the C57BL/6J male mice throughout the test, with no clear peak (Fig. 6c compared to Fig. 4a), suggesting a difficulty in the shift from investigation to interaction. When directly comparing the transitions made by BTBR and C57BL/6J male mice in the SP test, a significant difference was observed only during the last 2 min (Fig. 6d, strain: *F*_1,50_ = 7.870, *p* = 0.007; period: *F*_1,50_ = 15.079, *p* < 0.001; interaction: *F*_1,50_ = 3.874, *p* = 0.055, MM-ANOVA, post hoc *t* test *p* < 0.001). Accordingly, the investigation time of both stimuli was mainly spent on short bouts rather than on long bouts (Fig. 6e, stimulus: *F*_1,8_ = 6.789, *p* = 0.031; duration: *F*_3,24_ = 10.555, *p* < 0.001; interaction: *F*_3,24_ = 0.396, *p* = 0.757, TWR-ANOVA). Thus, it seems as if the BTBR mice display extended exploration phase and a reduced tendency for interaction with social stimuli.

The results of the SNP test showed very similar trends, with the exception of the transitions. As in the SP test, BTBR mice showed clear preference towards the novel social stimulus at the first 2 min (*p* < 0.05 paired *t* test after Bonferroni correction), but such a preference was not displayed during the last 2 min (Fig. 6f, g), similarly to female C57BL/6J mice (Fig. 2g). These results suggest a difference in the dynamics of social investigation between male BTBR and C57BL/6J mice in both the SP and SNP tests. Unlike the SP test, however, the dynamics of transitions in the SNP test did not seem to differ between the two strains, as BTBR mice also showed a high level of transition at early stages and a significant decline afterwards (Fig. 6h). Accordingly, no significant difference was found between the two strains in the total transition number either during the first or last 2 min of the SNP test (Fig. 6i; strain: *F*_1,49_ = 1, *p* = 0.322; period: *F*_1,49_ = 32.447, *p* < 0.001; *F*_1,49_ = 0.045, *p* = 0.833, MM-ANOVA, post hoc *t* test *p* < 0.001). Moreover, the distributions of investigation bouts according to bout duration in BTBR mice were qualitatively similar, although less significant (Fig. 6j; stimulus: *F*_1,7_ = 4.752, *p* = 0.066; duration: *F*_3,21_ = 3.195, *p* = 0.044; interaction: *F*_1.314,9.198_ = 2.347, *p* = 0.078, TWR-ANOVA) than in C57BL/6J male mice (Fig. 4e). These results suggest again that the SP and SNP tests probe distinct aspects of social behavior that may involve different dynamics. Altogether, these results suggest that our system may be used to probe differences in social behavior between various mouse strains.

## Discussion

A main goal of social neuroscience, one of the most rapidly developing fields in neuroscience, is to reveal and characterize the biological mechanisms underlying deficits in social behavior displayed by humans in pathological conditions such as ASD [1]. The use of animal models of such conditions seems to be crucial for the achievement of this aim [6–8]. Animal models enable examination and manipulation of biological mechanisms and serve to explore the effects of various interventions designed to correct their impaired social behaviors. Yet, efficient use of animal models requires standardization and automation of behavioral tests that will enable assessing social behavior in an unbiased manner, independently of the observer or the specific laboratory performing the experiments [9]. While such methods are widely used in other fields of neuroscience, they are hard to implement for exploration of the highly complex mammalian social behavior [10–12].

A breakthrough in this field was reported a dozen years ago by Moy et al. [2], who introduced the three-chamber test for the assessment of SP (sociability) and SNP. This test was shown to be efficient in revealing differences in social behavior between various strains of mice [13]. Since then, this test has become a standard procedure for assessing social behavior in mouse models of ASD [9, 14, 15].

Yet, despite several attempts to automate the three-chamber test [16, 17], it still suffers from several caveats: first, it measures the time spent by the subjects in each of the three compartments of the apparatus, rather than directly measuring social investigation behavior. While a good correlation was found between the two parameters in general [16], one cannot exclude the involvement of other behavioral parameters, such as spatial navigation and place preference, which may be independent of the motivation to investigate a specific social stimulus at certain conditions. Second, the test is mostly used in a manner which does not take in account the dynamics of the social behavior during the test (but see [18]). Third, the location of the stimulus in a round wire cage within its compartment creates difficulties to precisely relate specific investigation events with other measured parameters, such as vocalization or electrophysiological activity. Importantly, significant differences in both SP and SNP of several mouse strains were observed when either the time spent in the chamber or the time spent sniffing the stimulus were measured in the three-chamber test [13, 19]. It should be noted that several automated systems aiming to analyze social behavior in a higher resolution than the three-chamber test were recently published, each with its own advantages [20, 21].

Here, we presented a novel apparatus and analysis system that enable the same type of experiments for which the three-chamber apparatus is used, while solving the aforementioned caveats. The use of triangular chambers located in two corners of the arena restricts the area of interaction with the stimulus to an easily defined plane, which allows precise automated detection of investigation behavior events. This advantage is used by our open-source software to measure the dynamics of animal behavior in each of the employed tests. It also allows the random relocation of the chambers in opposite corners of the non-compartmentalized arena in each stage of the test, thus neutralizing any effect of spatial navigation, preference, or memory. These characteristics of our system enable direct assessment of the motivation for social investigation of each of the stimuli by the experimental subject. Using these advantages of the system, in combination with its ability to measure social behavior of subjects connected to cables, would allow recordings of physiological parameters during specific events of social investigation. Finally, the apparatus is compact, simple, and affordable, and the analysis software is publically available, making this system ready to use in any laboratory.

Using this system, we sought to characterize sex- and strain-specific differences in the SP and SNP tasks, for which the three-chamber test is widely used. We demonstrated, for the first time to our knowledge, that a main difference between male and female C57BL/6J mice in these tests is the dynamics of their behavior. In both tests, male mice were more persistent in their preference than females, which completely lost their preference towards one of the stimuli after 3 min of each test. It should be noted that no difference in SP or SNP between male and female C57BL/6J mice was observed by Moy et al. [2] using the three-chamber test. Nevertheless, they reported a significant reduction in the total time spent by females, as compared to males, in the social chambers during the SNP test. This observation is in agreement with our results, showing that females spent less time sniffing both social stimuli, as compared to males. Thus, by tracking the dynamics of social investigation using the system presented here, we were able to show previously identified and unidentified differences in social behavior between male and female mice.

Using our system, we compared two types of chambers, differing only in the level of separation between the subject and stimuli. While the grooved chambers enable rather limited social interactions, the meshed chambers allow better exposure of the social stimulus to the subject, with only a metal mesh separating between them. Whereas we found that both chambers can be efficiently used in the SP and SNP tests, the dynamics of social behavior is significantly different between them. Specifically, the meshed chambers seem to enhance longer bouts of social investigation towards the social stimulus, while reducing the number of short events, as compared to the grooved chambers. Since we found that most of the difference in investigation time between the stimuli was in longer bouts, we expect that the ability of our system to categorize investigation bouts according to their length will enhance the sensitivity of the preference analysis in the SP and SNP tests.

Using the meshed chambers, we analyzed the behavioral dynamics of male mice in both the SP and SNP tests and defined several new useful parameters, such as the duration of investigation bouts and number of transitions between stimuli. By analyzing these parameters, we found that both tests can be divided into initial exploration phase characterized by multiple transitions between stimuli and short investigation bouts, which is followed by interaction phase characterized by lower level of transitions and higher level of longer investigation bouts, mainly with the preferred stimulus. Interestingly, this dynamics was found to be altered in BTBR mice that showed high level of transitions and low level of long bouts throughout the SP test, suggesting a difficulty to shift from exploration to interaction. Notably, a similar reduction in the length of social interactions of BTBR mice as compared to C57BL/6J was recently reported using a different automated behavioral system [20]. Thus, the analysis of the behavioral dynamics in our system allowed us to define a new type of impaired social behavior in a mouse model of ASD. Further experiments with other types of ASD animal models will reveal if such impairment is a hallmark of their social behavior.

## Conclusions

To summarize, here, we present a novel design of a simple and affordable behavioral system that enables automated and precise measurements of social investigation behavior. Using the ability of this system to measure new parameters of social investigation, we demonstrated sex- and strain-specific differences in the dynamics of social behavior during the SP and SNP tasks. Thus, unlike the three-chamber test, our system provides multiple automatically measured parameters that enable a thorough analysis of the dynamics of social behavior. Such analysis should allow a detailed classification of mouse strains and genetically modified lines according to their specific social behavioral deficits. Moreover, the possibility of precise temporal detection of investigation bouts towards social stimuli demonstrated by us here, combined with the ability of the system to monitor the behavior of subjects connected to electrical cables or optical fibers, should enable using this system to record physiological parameters, such as brain neural activity, while the animals are investigating specific social stimuli. We believe that the system presented here would enhance the efforts to reveal the role of various brain networks and molecular mechanisms in mammalian social behavior and to examine their function in animal models of pathological conditions characterized by atypical social behavior, such as ASD.

## Methods

### Animals

Subjects were naïve C57BL/6J male and female mice (8–12 weeks), commercially obtained (Envigo, Israel) and housed in groups of three to five animals per cage. Stimuli were in-house grown juvenile male C57BL/6J mice (21–30 days old). Mice were kept on a 12-h light/12-h dark cycle, light on at 7 p.m., with ad libitum access to food and water. Behavioral experiments took place during the dark phase under dim red light. Stimuli mice were placed in the chambers for ~ 15 min prior to the experiment for acclimation. BTBR male mice (4–5 months old) were obtained from the animal facility of the Weizmann Institute (Rehovot, Israel) and held in similar conditions as above.

All experiments were approved by the Institutional Animal Care and Use Committee of the University of Haifa.

### Anesthesia

#### Surgery and implantation of electrodes

C57BL/6J male mice were head-fixed in a stereotaxic apparatus (Kopf Inst.) under isoflurane-mediated anesthesia, and a home-made tetrode probe (weighing 2 g) was inserted into the hypothalamus (*A*/*P* = − 0.85 mm, *L*/*M* = − 0.3 mm, *D*/*V* = − 4.8 mm) and fixed using dental cement. During experiments, the animals were connected to a recording system (RHD2000, Intan Technology) using an ultra-thin SPI interface cable (weighing 4.1 g, Intan Technology). The wire was hanged on a hook above the experimental setup.

#### Experimental setup

The experimental setup (Fig. 1a) consisted of a white Plexiglass arena (37 × 22 × 35 cm) placed in the middle of an acoustic chamber (60 × 65 × 80 cm). Two Plexiglass triangular chambers (12 cm isosceles, 35 cm height) were placed in two randomly selected opposite corners of the arena, in which animal or object (plastic toy) stimuli could be placed. Two versions of these chambers were used. In the first (grooved chambers), three horizontal slots (12 × 1 cm) were grooved one over the other (1 cm interval) at the bottom of the triangular chamber, thus enabling a subject rodent to investigate the stimulus in a restricted way through the slots. The second version (meshed chambers) was similar but employed a metal mesh (12 × 6 cm, 1 × 1 cm holes) placed instead of the slotted area, thus enabling direct interaction with the stimulus through the mesh. A high-quality monochromatic camera (Flea3 USB3, Point Grey), equipped with a wide-angle lens, was placed at the top of the acoustic chamber and connected to a computer, enabling a clear view and recording of the subject’s behavior using a commercial software (FlyCapture2, Point Grey).

#### Tracking software

To track the experimental subject and its interactions with the stimuli areas, four different algorithms were written in MATLAB (2015a-2016a), based on the image processing toolbox. The main goal of all algorithms was to track the boundaries of the subject body that are to be considered for evaluating the animal’s direct contact with the “stimuli” areas.

The basic algorithm tracks the boundaries of an unwired dark mouse (body-based algorithm) on a light background. The graphical user interface (GUI; Additional file 1) of the software includes the ability to set up the threshold for animal detection, thus enabling fine-tuning of the detection.

The second (head directionality-based) algorithm is based on the body-based algorithm, with the addition of head directionality evaluation. Using this algorithm, we could evaluate the subject’s head interactions with the “stimuli” areas, thus avoiding false interactions caused by random contact of rest of the body with the “stimuli” areas. In the GUI, the experimenter defines two detection thresholds of mouse body boundaries: high, which includes the brighter tail of C57BL/6 mice, and low, which does not. Later on, the algorithm fits an ellipsoid to the boundaries detected using the lower threshold, from which an estimation of the mouse head and tail locations is calculated (with no distinction between the two). The final determination of the tail and head is done based on the boundaries of the higher threshold.

The third (wired body-based) algorithm is also based on the body-based algorithm but with additions aiming to minimize artifacts resulting from cables (either electrical wire or optical fiber) connected to the animal.

All variations of the software are deposited in GitHub under the following link: https://github.com/shainetser/TrackRodent

#### Behavioral paradigm

The behavioral paradigm consisted of a 20 min open-field test, followed by insertion of the subject animal to an empty chamber and 15 min of habituation. Thereafter, social and object stimuli were randomly inserted each to a distinct chamber, and the SP test was performed for 5 min. Following the SP test, the chambers with the stimuli were removed from the arena, and the subject was left alone for 15 min. Then, the chambers were inserted again, this time to the other two corners of the arena, with one containing the same social stimulus used for the SP test (familiar stimulus) and the other containing a novel stimulus, and the SNP test took place for 5 min. Notably, the familiar stimulus was always placed in a different corner relative to the SP test. At the end of the SNP test, the experimental subject was placed back in its home cage, while the stimuli were left in the chambers for the next experiment or placed back in their home cage at the end of the experimental session.

For experiments with wired mice, the animals were placed in the arena with empty chambers for 25 min of habituation with the electrophysiological system wired to the probe connector located on their heads. Then, one of the empty chambers was replaced by one with a social stimulus for 5 min (marked by a dashed line in Fig. 6f).

#### Analysis

All analyses were done after correcting the raw behavioral data by neglecting any gap of < 15 frames (0.5 s) in investigation of a given stimulus, and not considering it as breaking the investigation bout. Investigation time was basically calculated within 20-s bin across the 5 min SP and SNP tests. In some analyses, we extracted the different investigations bouts according to their length and summed them for calculation of investigation time for each duration category.

RDI was defined as the absolute value of the difference between the investigation durations towards the two stimuli, divided by the sum of them.

Intervals between investigations were defined as time gaps between investigations towards the same stimulus that are larger than 0.5 s.

Transitions between stimuli were defined as the time points when investigation of a new stimulus (relative to the other stimulus) started.

Distance traveled during the open-field test was defined as the total distance traveled by the center of the body.

Center/periphery ratio was defined as the time spent in the inner quarter of the arena divided by the time spent in the outer three quarters, throughout the open-field test.

#### Statistics

All statistical tests were performed using SPSS v21.0 (IBM). Kolmogorov-Smirnov and Shapiro-Wilk tests were used for normality check. A one-tailed paired *t* test was used to compare between different conditions or stimuli for the same single group, and a one-tailed independent *t* test was used to compare a single parameter between distinct groups. For comparison between multiple groups and parameters, a mixed analysis of variance model (MMM-ANOVA) was applied to the data. This model contains one random effect (ID), one within effect, one between effect, and the interaction between them. For comparison within a group using multiple parameters, a two-way repeated measures analysis of variance model (TWR-ANOVA) was applied to data. This model contains one random effect (ID), two within effects, and the interaction between them. All ANOVA tests were followed, if main effect or interaction found, by post hoc Student’s *t* test with Bonferroni correction. Significance was set at 0.05 and was adjusted when multiple comparisons used.

## Additional files

Additional file 1: Graphical user interface (GUI) of the software. A PDF file showing a picture of the GUI. (PDF 430 kb)

Additional file 2: Analysis of the video using body-based algorithm. A short video clip (AVI file) showing how the software analyze the mouse investigation behavior using the body-based algorithm. The white cross reflected by the software on the animal’s body changes its color according to the stimulus (blue or green) in each frame the software detects as an event of stimulus investigation. (AVI 6618 kb)

Additional file 3: Analysis of the video using head directionality-based algorithm. A short video clip (AVI file) showing how the software analyze the mouse investigation behavior using the head directionality-based algorithm. The white diamond reflected by the software on the animal’s head changes its color to yellow in each frame the software detects as an event of stimulus investigation. (AVI 6211 kb)

Additional file 4: Behavioral dynamics of male and female mice—time in compartments. A PDF file showing the behavioral dynamics of male (A, C) and female (B, D) mice in the SP (A, B) and SNP (C, D) tests, as measured from the time they spend in each virtual compartment (half of the arena). (PDF 339 kb)

Additional file 5: Repeatability of behavioral results. A PDF file depicting the behavioral dynamics of two experimental groups of male (left) and female (right) mice. Each group (*n* = number of animals in each group) comprises animals that were tested together in the same time (one to two consecutive days). (PDF 343 kb)
