# Supplementary material for: A novel system for tracking social preference dynamics in mice reveals sex- and strain-specific characteristics
Source: Mol Autism. 2017 Oct 3;8:53. doi: 10.1186/s13229-017-0169-1 (PMC5627457; doi:10.1186/s13229-017-0169-1)
Supplement: Supplementary file 1 — Graphical user interface (GUI) of the software. A PDF file showing a picture of the GUI. (PDF 430 kb) [file 13229_2017_169_MOESM1_ESM.pdf]

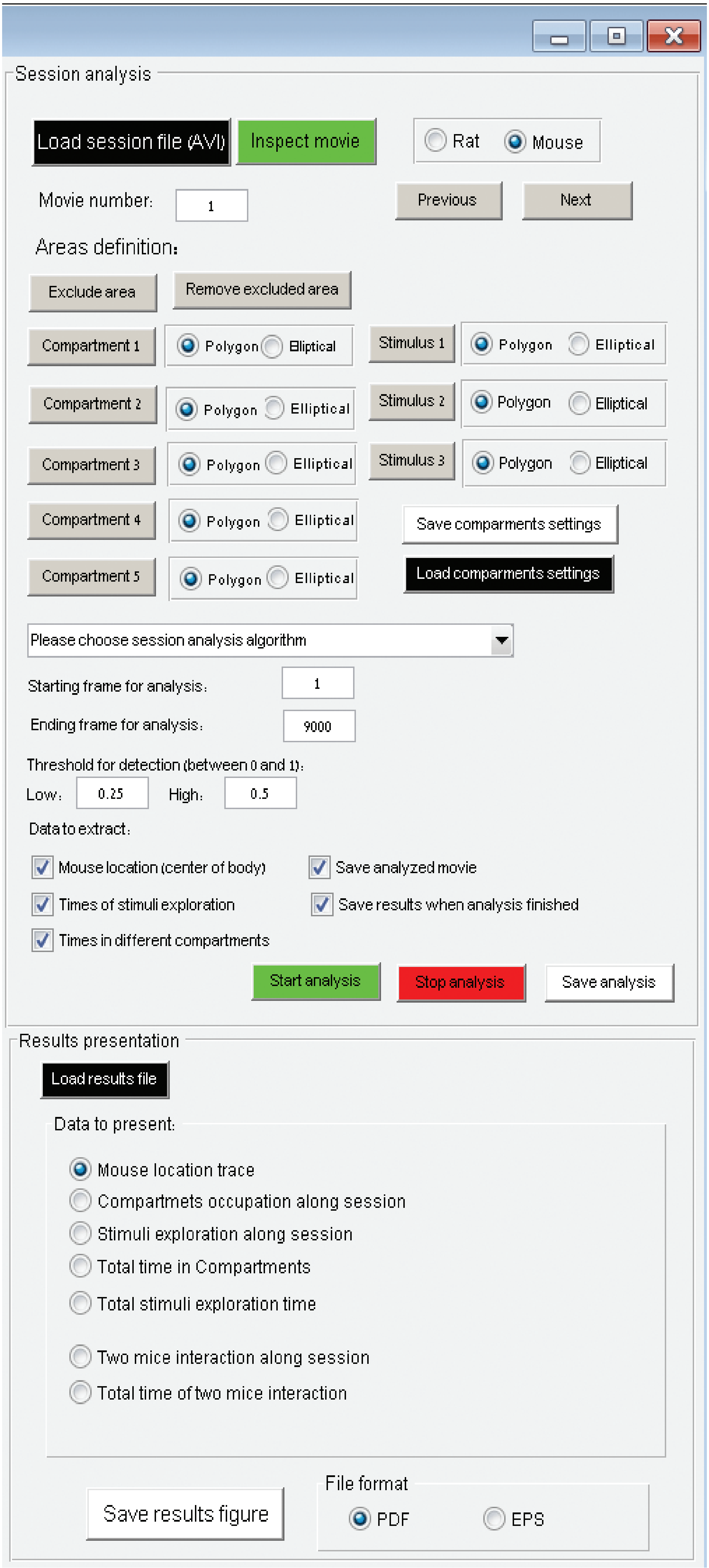
Session analysis
Load session file (AVI)
Inspect movie
Rat
Mouse
Movie number:
1
Previous
Next
Areas definition:
Exclude area
Remove excluded area
Compartment 1
Polygon
Elliptical
Stimulus 1
Polygon
Elliptical
Compartment 2
Polygon
Elliptical
Stimulus 2
Polygon
Elliptical
Compartment 3
Polygon
Elliptical
Stimulus 3
Polygon
Elliptical
Compartment 4
Polygon
Elliptical
Save comparments settings
Compartment 5
Polygon
Elliptical
Load comparments settings
Please choose session analysis algorithm
Starting frame for analysis:
1
Ending frame for analysis:
9000
Threshold for detection (between 0 and 1):
Low:
0.25
High:
0.5
Data to extract:
Mouse location (center of body)
Save analyzed movie
Times of stimuli exploration
Save results when analysis finished
Times in different compartments
Start analysis
Stop analysis
Save analysis
Results presentation
Load results file
Data to present:
Mouse location trace
Compartmets occupation along session
Stimuli exploration along session
Total time in Compartments
Total stimuli exploration time
Two mice interaction along session
Total time of two mice interaction
Save results figure
File format
PDF
EPS
