# Supplementary figures and images for: A novel system for tracking social preference dynamics in mice reveals sex- and strain-specific characteristics
Source: Mol Autism. 2017 Oct 3;8:53. doi: 10.1186/s13229-017-0169-1 (PMC5627457; doi:10.1186/s13229-017-0169-1)

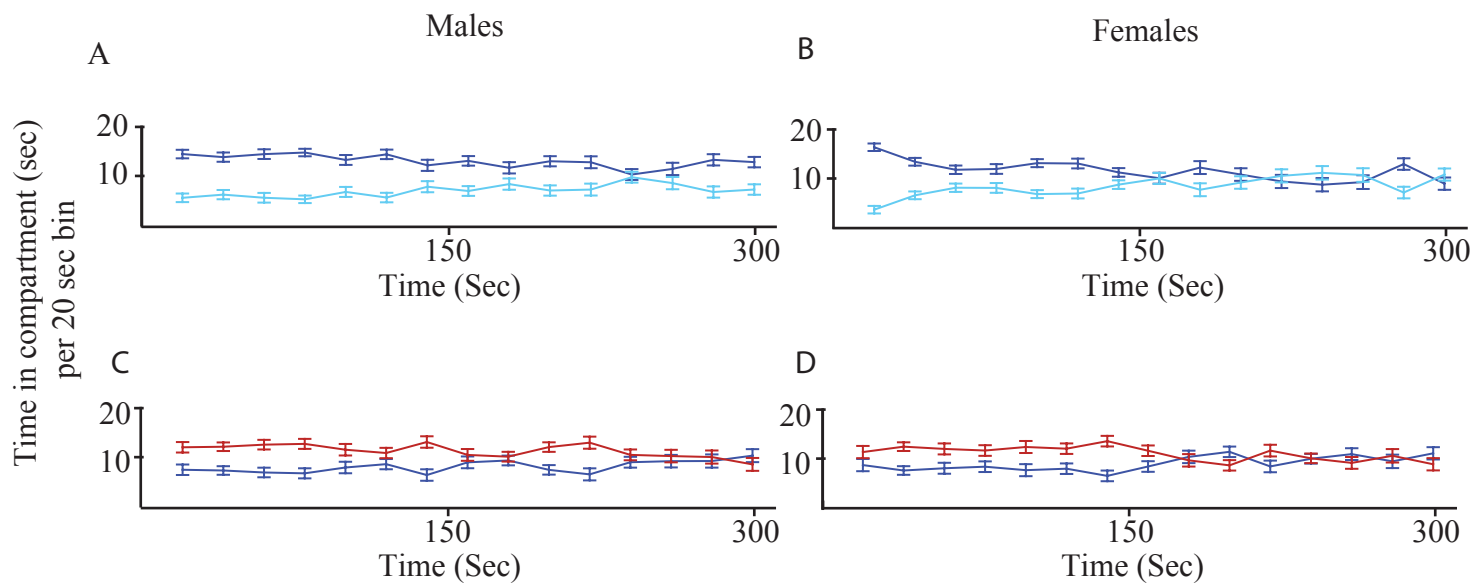

Supplement: Supplementary file 4 — Behavioral dynamics of male and female mice—time in compartments. A PDF file showing the behavioral dynamics of male (A, C) and female (B, D) mice in the SP (A, B) and SNP (C, D) tests, as measured from the time they spend in each virtual compartment (half of the arena). (PDF 339 kb) [file 13229_2017_169_MOESM4_ESM.pdf]

Males

Group 1 - n=8

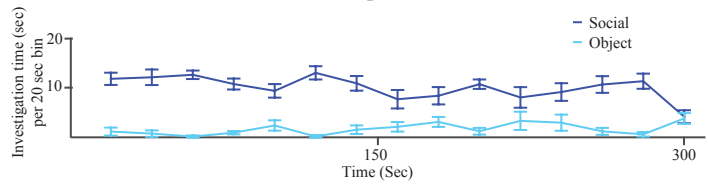

Females

Group 1 - n=12

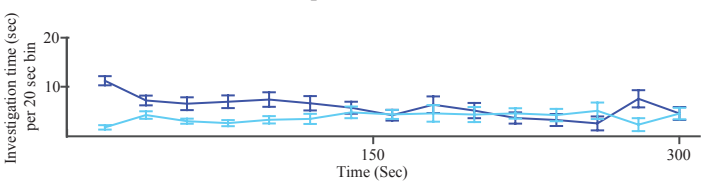

Group 2 - n=15

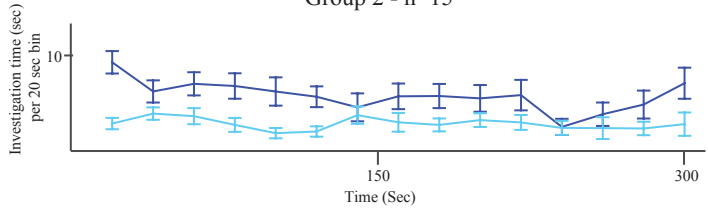

Group 2 - n=10

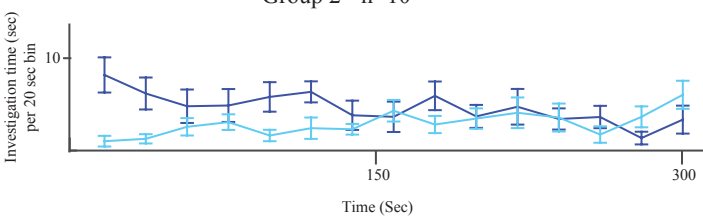

Supplement: Supplementary file 5 — Repeatability of behavioral results. A PDF file depicting the behavioral dynamics of two experimental groups of male (left) and female (right) mice. Each group (n = number of animals in each group) comprises animals that were tested together in the same time (one to two consecutive days). (PDF 343 kb) [file 13229_2017_169_MOESM5_ESM.pdf]
